# New treatment option for an incomplete vertical root fracture–a preliminary case report

**DOI:** 10.1186/1746-160X-10-9

**Published:** 2014-03-26

**Authors:** Paul Henryk Hadrossek, Till Dammaschke

**Affiliations:** 1 Department of Operative Dentistry, Albert-Schweitzer-Campus 1, Building W 30, Waldeyerstr. 30, 48149 Münster, Germany

**Keywords:** Biodentine, Calcium silicate cement, MTA, Treatment, Vertical root fracture

## Abstract

Instead of extraction this case report presents an alternative treatment option for a maxillary incisor with a vertical root fracture (VRF) causing pain in a 78-year-old patient. After retreatment of the existing root canal filling the tooth was stabilized with a dentine adhesive and a composite restoration. Then the tooth was extracted, the VRF gap enlarged with a small diamond bur and the existing retrograde root canal filling removed. The enlarged fracture line and the retrograde preparation were filled with a calcium-silicate-cement (Biodentine). Afterwards the tooth was replanted and a titanium trauma splint was applied for 12d. A 24 months clinical and radiological follow-up showed an asymptomatic tooth, reduction of the periodontal probing depths from 7 mm prior to treatment to 3 mm and gingival reattachment in the area of the fracture with no sign of ankylosis. Hence, the treatment of VRF with Biodentine seems to be a possible and promising option.

## Background

Vertical root fractures (VRF) are fractures of enamel and dentine along the long axis of the tooth towards the apex [1,2]. Besides trauma, a VRF may be caused due to weakening of the dental hard tissue during root canal treatment or restoration, placement of posts and pins, parafunctional habits, heavy stressful chewing and occlusal overload [1-4]. Unfortunately, VRF are serious complications with poor prognosis. Hence, in case of a confirmed diagnosis, therapy of VRF is extraction [1-5]. Main factors which lead to extraction of a fractured tooth are bacterial infiltration causing subsequent inflammation in the fracture area, as well as resorption of nearby alveolar bone induced by defensive cells [6]. Nevertheless, in the past, a variety of approaches have been made to treat VRF, e.g. with cyanoacrylates [7], glass ionomer cement in combination with guided tissue regeneration [8], adhesive composite resins [9-16] and Mineral Trioxide Aggregate (MTA) [4,17-19].

But so far none of these treatment options provided ideal long term results [13]. Thus, these treatment options are more or less of temporary nature as long-term success rates are considerably low [20,21]. Even with MTA the clinical results were disappointing and the attempt to preserve teeth with VRF by using MTA was rejected [4]. Hence, until today, no valid treatment option to preserve teeth with VRF can be recommended. Nevertheless, in this case report for the first time a new treatment option for a maxillary incisor with a VRF is presented. By using a new calcium silicate cement (Biodentine) this fractured tooth could be kept in situ for an observation period of two years - free of any complains until now.

## Case presentation

**Figure 1:**
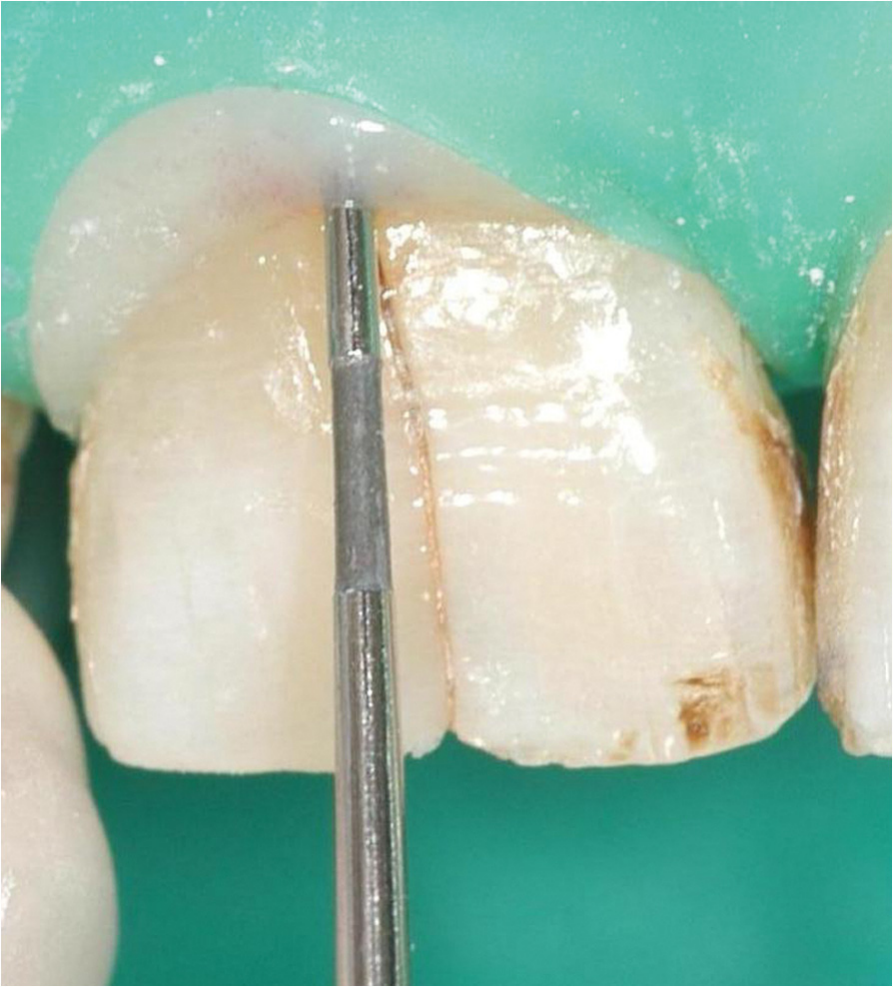
Fracture line on the labial surface of tooth 11 and periodontal probing depth of 7 mm in the area of the VRF.

A 78-year-old patient was referred to the Department of Operative Dentistry because her right central incisor (tooth 11) caused pain for the last 2 years after traumatic injury. Because of the continuous discomfort the tooth had already undergone an endodontic treatment including an apicoectomy after the trauma. On clinical examination the tooth showed a dark coloured fracture line on the labial surface (Figure 1). Furthermore, it showed positive rebound tenderness. The periodontal probing depth adjacent to the fracture line was 7 mm while the other probing depths showed a non-pathologic value of 2 - 3 mm (Figure 1).

Actually extraction was the only treatment option for this vertically root fractured tooth followed by an implant, a bridge or a dental prosthesis. None of these treatment options found acceptance by the patient (because of financial or comfort issues). So an alternative and new idea of treating the VRF was discussed, which the patient endorsed.

**Figure 2:**
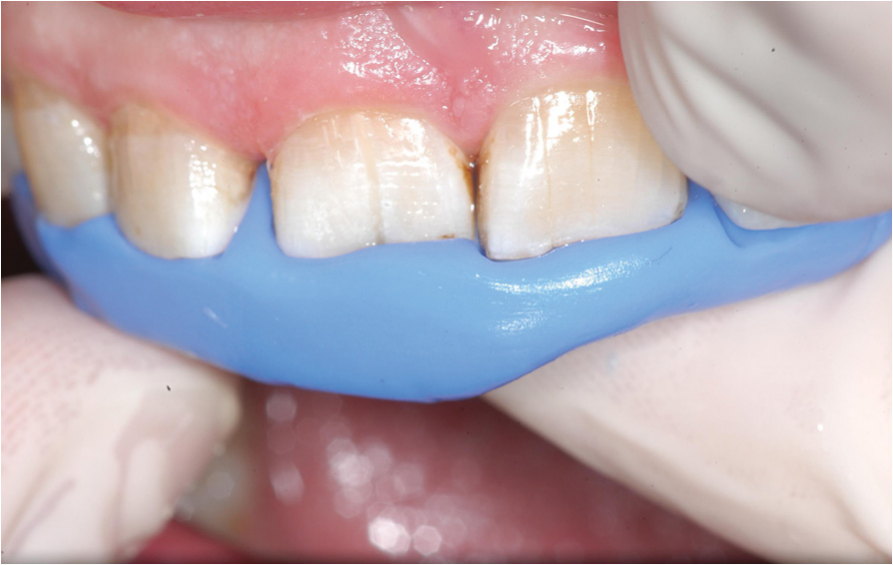
Restored coronal fracture line and silicone key prior to intentional extraction.

**Figure 3:**
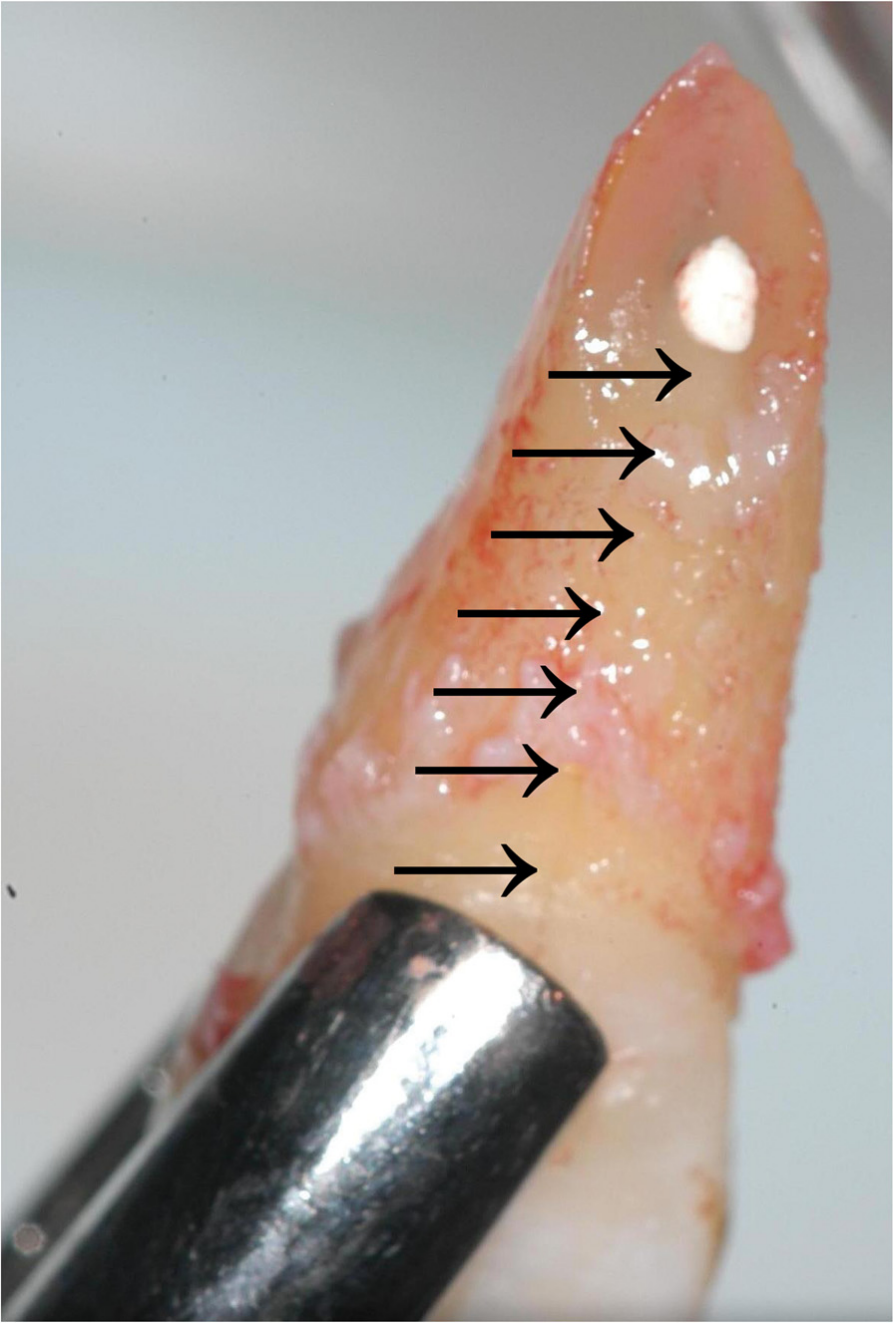
Extracted tooth 11 with fracture line (arrows) on the labial surface.

**Figure 4:**
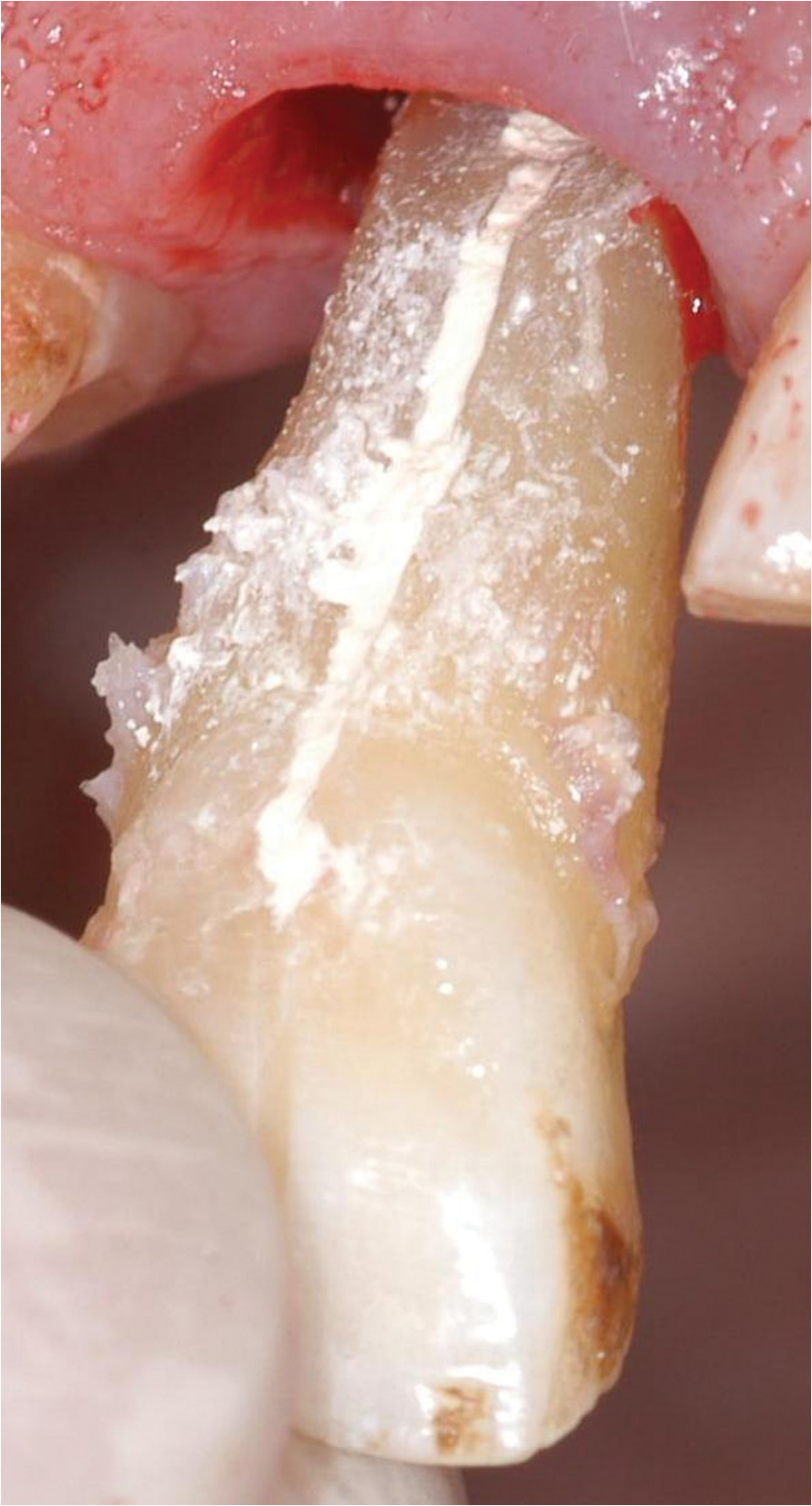
Enlarged fracture line filled with Biodentine (Septodont, St. Maur, France) prior to replantation.

**Figure 5:**
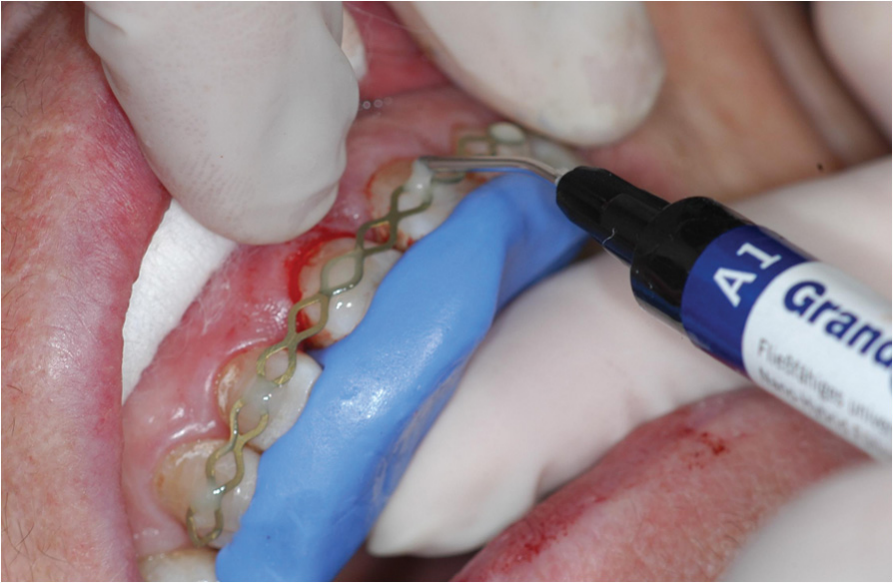
Replanted tooth 11 with silicone key for correct reposition and application of the titan trauma splint (TTS; Medartis, Basel, Switzerland).

**Figure 6:**
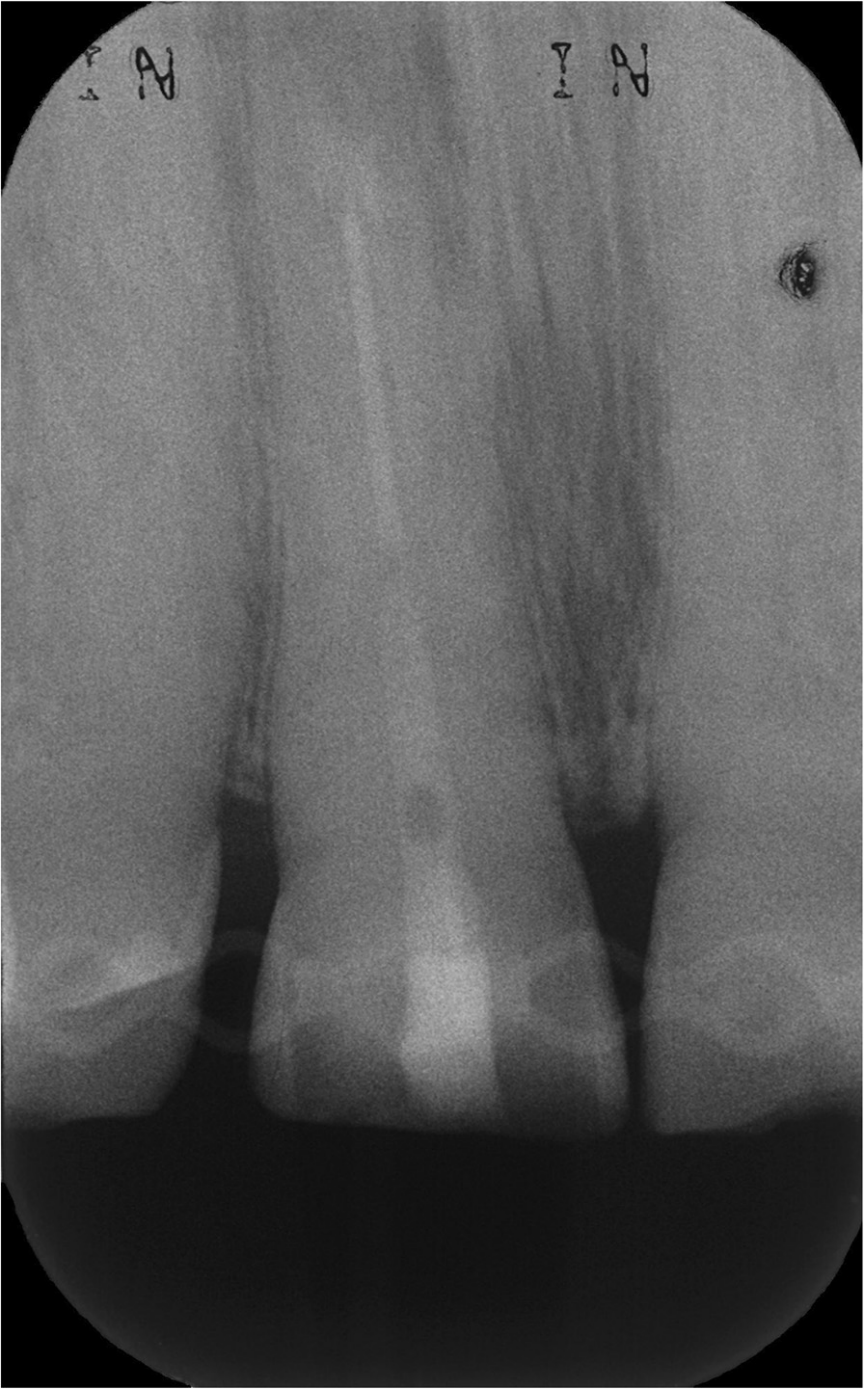
Control radiograph of tooth 11 after replantation.

**Figure 7:**
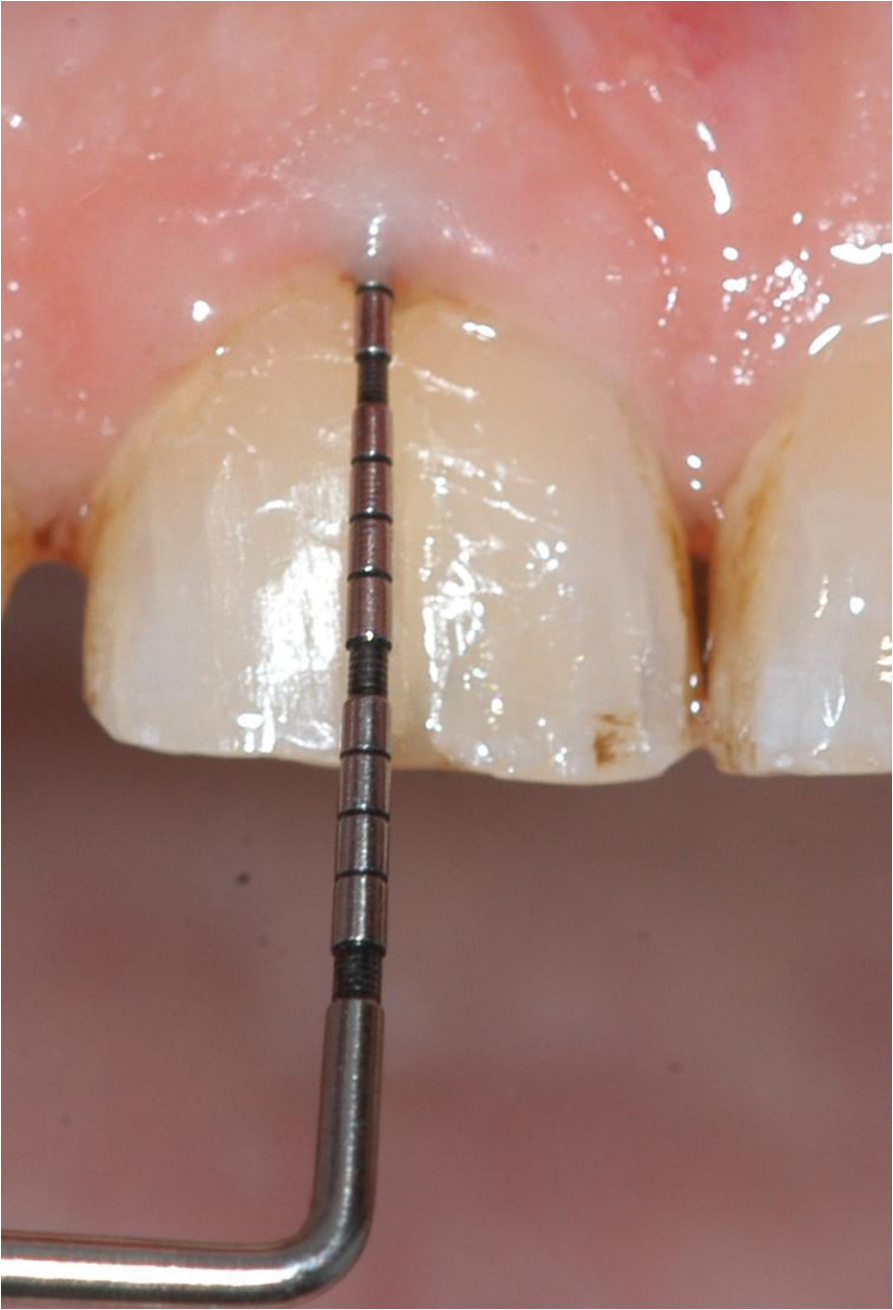
Periodontal probing depth of 3 mm in the area of the VRF 3 months after operation.

**Figure 8:**
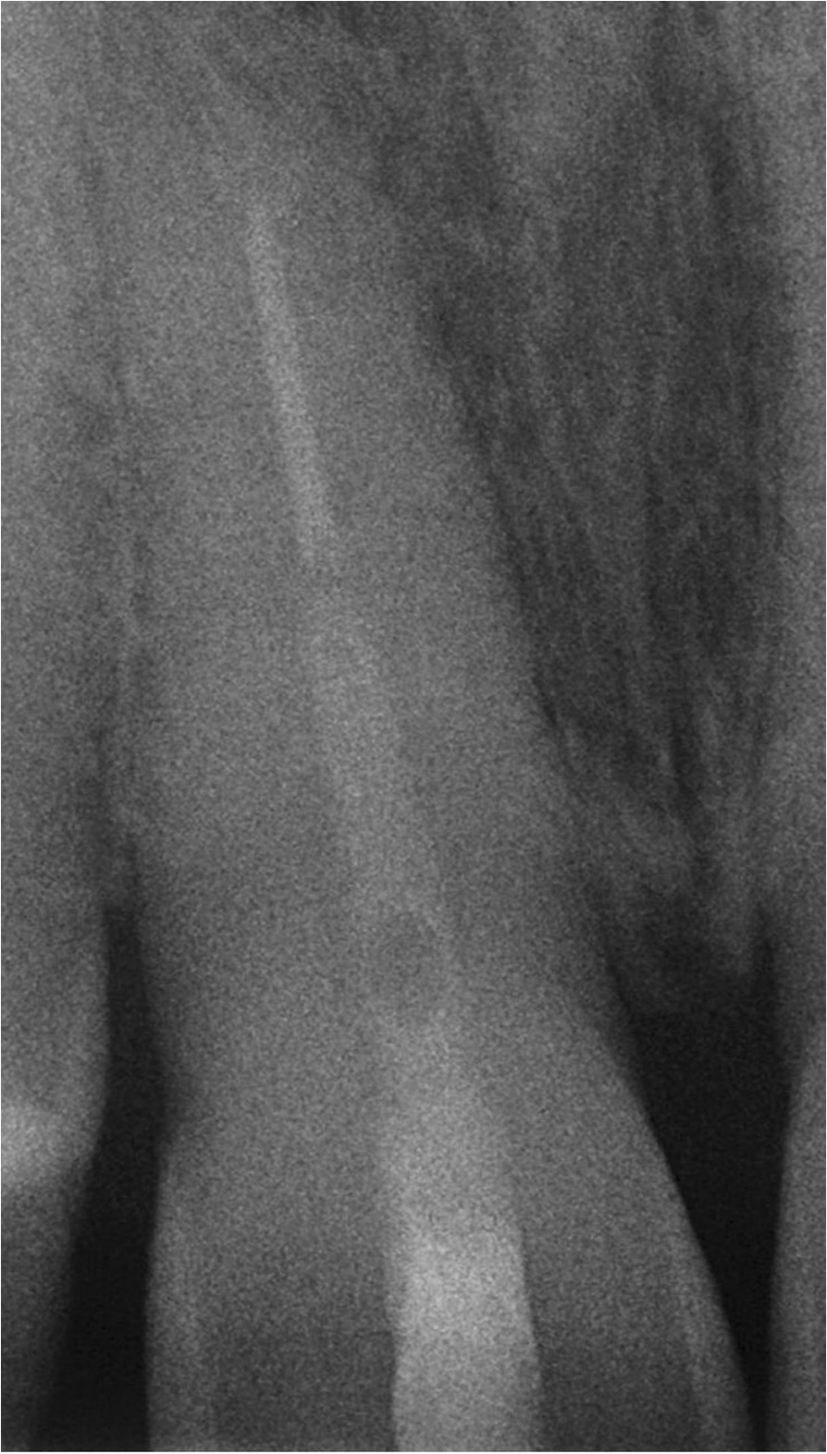
Control radiograph of tooth 11 24 months after replantation.

Under rubber dam isolation the coronal fracture line was enlarged with a small diamond bur. Additionally, 2/3 of the existing root canal filling was removed. The tooth was then stabilized coronally and intracanallary with a dentine adhesive (OptiBond All-In-One; Kerr, Orange, USA) and a composite restoration (Grandio/Grandio Flow; VOCO, Cuxhaven, Germany) (Figure 2). After removing the rubber dam and producing a silicon key (Figure 2), a titanium trauma splint (TTS; Medartis, Basel, Switzerland) was adapted and the area was anaesthetized with Ultracain D-S (Sanofi Aventis, Frankfurt, Germany). Tooth 11 was extracted very carefully to protect the surrounding hard and soft tissues and stored in and cleaned with the solution of a Dentosafe tooth rescue box (Medice Pharma, Iserlohn, Germany). While the palatinal surface of the root was unimpaired, the labial surface showed a vertical fracture line (Figure 3). It was enlarged with a small diamond bur and the existing retrograde root canal filling was removed. The enlarged fracture line and the retrograde preparation were filled with Biodentine (Septodont, Saint Maur, France) (Figure 4). While waiting for the Biodentine to set initially, the remaining root surface was constantly rewetted with the Dentosafe solution. Afterwards the tooth was replanted and the titanium trauma splint was applied for 12 d (Figure 5). The control radiograph showed a successfully replanted tooth 11 (Figure 6). After 3 months a clinical and radiological follow-up already presented an asymptomatic tooth, reduced periodontal probing depths from 7 mm prior to treatment to 3 mm and gingival reattachment in the area of the fracture (Figure 7). These findings were identically in the follow-ups after 6, 12 and 24 months. The percussion test sounded normal and there was no sign of ankylosis over the whole period of time. The dental film recorded 24 months after intentional extraction and replantation does not show any pathological findings on tooth 11 (Figure 8).

## Discussion

So far treatment options for VRF are very limited. It is a complication, which leads to extraction in nearly every case [1-5]. Hence, in the present case, extraction of tooth 11 and replacement by implant, bridge or denture was discussed with the patient. But all suggested treatment options after extraction were neither affordable for the patient (implant), nor favoured because of aesthetic properties (bridge) or restricted wearing comfort (denture). Thus, an alternative treatment planning to restore the VRF was required and the use a calcium silicate cement was suggested.

Since its introduction as endodontic reparation cement ProRoot MTA (Dentsply Tulsa, Tulsa, USA) has been examined in several studies; it is meanwhile well accepted and probably the most established material for the treatment of e.g. root-end fillings, apexification or closure of radicular perforations and other dentine defects in the root [22-24]. Hence, the attempt to repair VRF with MTA has been described previously [4,17-19]. Even though ProRoot MTA has many positive features, it also has several drawbacks: difficult handling, long setting time, possible discoloration if used in the visible crown area, lower compressive and flexural strength than dentine (should not be used as a restorative base) and its high costs [25-28]. Thus, over the last years, several calcium silicate cements comparable to MTA have been presented, which can be used as endodontic repair material. One of these materials is Biodentine. It is a bioactive cement mainly consisting of tri- and dicalcium-silicate. When using Biodentine in the repair of VRF it may have some advantages compared to MTA. At first the initial setting time is about 15 min [28,29]. Even though Grech et al. evaluated the definite setting time of Biodentine to be 45 min (according to ISO 9917-1:2007) [30], this is much faster than the mean setting time for ProRoot MTA with 165 ± 5 min [31]. Fast setting time is important for the suggested treatment option to keep the extraoral time short to avoid drying of the PDL cells and to provide a resistant filling when replanting the tooth.

Another positive feature of Biodentine is that its Vickers micro hardness (HV) is approximately 60 HV [32] which is similar to dentine. The HV value of sound human dentine was reported to be between 60 HV and 90 HV [33,34] while the HV for ProRoot MTA was determined to be about 40 HV [35].

According to the manufacturer’s information an important advantage of Biodentine is its resistance to hydrolysis while setting. Thus, it could be used as a temporary filling material because it doesn’t dissolve in contact with saliva [28,29,36].

Further advantage is its biocompatibility. Biodentine showed significantly higher levels of calcium and silicon ion release than MTA [37,38]. Especially silicon plays an important role in the bioactivity of Biodentine. It increases bone calcification [39,40] and stimulates bone growth [41-43]. It also has a positive effect on the mineralization of dentine [44].

Furthermore, Biodentine is - identically to MTA - able to develop a hydroxyl apatite-like surface in the presence of body liquids containing calcium or phosphate [45]. This surface is biocompatible and displays good conditions for cell attachment and proliferation of the PDL [37,45]. In how far Biodentine had a positive influence on the regeneration of PDL cells cementogenesis and other reparative procedures after intentional extraction followed by replantation can not be evaluated from this clinical case report but need to be studied in further research.

In the present case the tooth 11 was intentionally extracted and replanted, after repair of the VRF with Biodentine. An alternative to the extraction would have been the repair of the VRF by surgical exploration, which has been described in the literature as a possible treatment before [4,8,17-19]. This treatment includes dissecting a flap to visualize the bone loss and providing the fracture line with different materials [4,8,17-19].

A disadvantage is that this procedure causes a scar in the visual area of the gingiva. For exploring the extent of the fracture line and its treatment additional osteotomy is necessary which generates extra loss of healthy bone structure. A gingival recession can be expected, so in some regions this procedure is not indicated because of aesthetic considerations [46,47].

Intentional extraction and replantation of a tooth in order to manage VRF has already been described in literature [7,9-16]. Contraindications are teeth, which probably cannot be extracted and repositioned due to a complicated root anatomy, teeth with a severe periodontitis, teeth without adjacent teeth, a non-compliant patient and patients with critical general medical conditions. Before extraction pretreatment of the tooth is necessary. Amalgam or temporary non-adhesive fillings should be removed. Root canal treatment needs to be done prior to the extraction in order to reduce the extraoral time. Approximately two third of the root canal filling should be removed to stabilize the tooth within the root and crown by applying a composite restoration.

In order to preserve the PDL cells an infiltration anaesthesia was preferred over an intraligamentary anaesthesia. The extraction itself should be done as conservative as possible. After loosening the gingiva and the PDL cells at the crestal level with a periotome or a micro-scalpel, the tooth can be slightly loosened out of its socket with a lever. The dental cement and periodontium should be protected as good as possible. When using a forceps, the branches should not exceed the cement-enamel junction. Defects on the cement layer could lead to resorptions or ankylosis [46]. All defect preparation and removing of the retrograde filling should be done under constant cooling with isotonic saline solution.

A short extraoral time of the tooth seems to be very important regarding a positive healing tendency. Drying for an extensively long time would lead the PDL cells to die off, what significantly raises the possibility of root resorptions [48,49]. The extraoral time of the tooth after extraction was less than 25 minutes. Within the procedures the root surface was repetitively rewetted with Dentosafe solution.

After treatment of the fracture line with Biodentine and removing the blood clot from the socket with saline rinse, the tooth should be repositioned pressureless and fixed with a titanium trauma splint for approximately 10 days [46]. Longer splinting could lead to ankylosis or resorption [46,50,51].

A systemic administration of antibiotics is not necessary. Even though experimental studies have shown, that its use can decrease the possibility of root resorptions, a clinical report with 400 replanted teeth could not support this effect [52]. Since the extraction and replantation was conducted under ideal clinical conditions, the topical use of antibiotics or dexamethasone as suggested for avulsed teeth after traumatic injuries [48] can also be abstained. The bacterial contamination of the root surface under clinical conditions can be considered to be much lower than after avulsion as a result of an accident. In order to minimize the bacterial content in the oral cavity prior to treatment, the patient should be instructed to use 0.1% to 0.2% chlorhexidine oral rinse twice a day, one day before the procedure as well as right before and afterwards. Furthermore, an ideal oral hygiene is mandatory.

## Conclusion

It must be kept in mind that this is a single case study and the observation period of two years is quite short. Thus, it is difficult to extrapolate a single case to a more general conclusion. For a general recommendation, whether this is a suitable treatment option for VRF, more cases over a longer period of time need to be monitored. Nevertheless, intentional extraction and filling the fracture gap with Biodentine followed by replantation is a new clinical treatment option for teeth which have to be extracted elsewise. Hence, the described treatment may contribute to change the clinical practice of VRF in future.

## Consent

Written informed consent was obtained from the patient for publication of this Case report and any accompanying images. A copy of the written consent is available for review by the Editor-in Chief of this journal.

## Competing interests

The authors declare that they have no competing interests.

## Authors’ contributions

Both authors have contributed significantly to this work and contributed to the paper in the equal parts: PHH had the idea for the treatment and developed the concept, participated in literature research and writing of the manuscript. TD participated in literature research and writing of the manuscript and carried out proofreading. Both authors read and approved the final manuscript.
